# Crimean-Congo hemorrhagic fever virus localization and shedding in the reproductive tract of lethal and survivor mouse models

**DOI:** 10.1080/22221751.2025.2595795

**Published:** 2025-11-27

**Authors:** Teresa E. Sorvillo, Jana M. Ritter, Stephen R. Welch, Katherine A. Davies, JoAnn D. Coleman-McCray, Heather M. Hayes, Georgia Ficarra, Julu Bhatnagar, Scott D. Pegan, Éric Bergeron, Joel M. Montgomery, Christina F. Spiropoulou, Jessica R. Spengler

**Affiliations:** a CDC Foundation assigned to Viral Special Pathogens Branch, Division of High-Consequence Pathogens and Pathology, Centers for Disease Control and Prevention, Atlanta, GA, USA; b Infectious Diseases Pathology Branch, Division of High-Consequence Pathogens and Pathology, Centers for Disease Control and Prevention, Atlanta, GA, USA; c Viral Special Pathogens Branch, Division of High-Consequence Pathogens and Pathology, Centers for Disease Control and Prevention, Atlanta, GA, USA; d Division of Biomedical Sciences, University of California Riverside, Riverside, CA, USA

**Keywords:** Crimean-Congo hemorrhagic fever virus, mice, viral persistence, sexual transmission, reproductive tract, hemorrhagic fever virus

## Abstract

Hemorrhagic fever viruses have been shown to localize to immune-privileged sites, including the reproductive tract, raising important questions about long-term persistence and the potential for sexual transmission. Anecdotal evidence of sexual transmission of Crimean-Congo hemorrhagic fever virus (CCHFV) has been reported, and *in vivo* studies suggest that CCHFV can localize to reproductive tissues; however, to date, this phenomenon has not been explicitly investigated. We evaluated histopathology and viral loads (viral RNA, viral antigen, and infectious viral titres) in reproductive tissues obtained from lethal and survivor mouse models of CCHFV during acute and convalescent phases of infection. Viral loads in urogenital swabs were also evaluated to assess the potential for virus transmission. Although no evidence of long-term persistence was observed in the survivor model of CCHF, our data indicate a potential for sexual transmission during acute infection, even in cases of mild disease, as infectious virus was isolated from urogenital swabs. These data support the importance of sampling human patients to better define the risk of sexual transmission and potential viral persistence in reproductive tissues during and after recovery from CCHF.

## Introduction

Crimean-Congo hemorrhagic fever virus (CCHFV) is estimated to infect 10,000 to 15,000 people each year with three billion people believed to be at risk of infection [1]. CCHFV can cause a wide spectrum of human clinical disease where fatality occurs in approximately 30% of cases. Most frequently it causes mild-to-moderate illness where individuals recover from acute infection and reach convalescence [2]. Data suggest that subclinical infections also occur, with some studies estimating the incidence to be as high as 88% of total infections [3]. Despite the large number of individuals who survive infection, information remains limited regarding long-term sequelae and virus persistence in survivors. Historically, persistence of hemorrhagic fever viruses in immune-privileged tissues such as the eye [4] and reproductive tract [5,6], has been underrecognized. However, in recent years, relapse of acute Ebola virus (EBOV) disease following convalescence has been documented [7], and Lassa virus has been shown to persist and intermittently shed from the reproductive tract of human survivors [8]. Further, reports of sexual transmission of EBOV from convalescent individuals [9] highlight that viral tropism and persistence in reproductive tissue can have significant public health consequences, including the initiation of new outbreaks [6,10,11].

The possibility of CCHFV sexual transmission has been reported in two publications. The first in 2013 describes a patient who had sexual intercourse with his wife after resolution of acute CCHFV infection; his wife developed CCHF 11 days after he was discharged from the hospital and 7 or 8 days after sexual contact [12]. The second report from 2016 describes three confirmed cases of CCHF where the only known exposure was sexual contact with an infected spouse either before their symptom onset (2 cases) or after improvement of acute symptoms (1 case) [13]. Additionally, two reports of epididymo-orchitis as a complication of CCHF [14,15] and persistence of CCHFV viral RNA (vRNA) in the endometrial tissue of a pregnant individual [16] demonstrate a potential tropism of the virus for reproductive tissues in humans. In animal models, CCHFV persistence has been documented in the testes of nonhuman primates; however, co-infection with tuberculosis makes these results difficult to interpret [17].

We previously reported pronounced tropism of a fluorescent CCHFV reporter virus for reproductive tissues after lethal infection in female IFNAR^-/-^ mice [18]. Here, we expand on this work by characterizing tissue tropism, replication kinetics, histopathology, and viral shedding from the reproductive tracts of male and female mice using lethal and survivor models [19]. By examining acute and convalescent phases of infection, these studies provide a framework for understanding the potential risk of sexual transmission across different stages and severities of CCHF.

## Materials and methods

### Biosafety and ethics statement

Studies involving infectious CCHFV were carried out at the Centers for Disease Control and Prevention (CDC; Atlanta, GA) in Biosafety Level 4 (BSL-4) containment facilities. Experiments involving recombinant viruses were conducted under protocols approved by the CDC Institutional Biosafety Committee (IBC). All animal research was approved by the CDC Institutional Animal Care and Use Committee (IACUC; #3102, 3342, 3343) and performed in an AAALAC-International accredited facility.

### Viruses and cells

The detailed passage history of CCHFV strains IbAr10200 (GenBank: KJ648914, KJ648915, KJ648913; VirHARV #813730) and Turkey04 (GenBank: KY362517, KY362519, KY362515; VirHARV #813732) was described previously [20]. HuH-7 cells were sourced from Apath, LLC.

### Mice

C57BL/6J mice (The Jackson Laboratory, strain #000664; male and female) were housed as previously described [19]. Groups of mice were immunosuppressed via intraperitoneal (IP) injection with anti-IFNAR1 monoclonal antibody (mAb) MAR1-5A3 (2.5 mg administered −1/+1, 0, or +1 dpi; Leinco Technologies, Cat. No. I-401, Lot Nos. 1223L560, 0823L230, 0822L285, 0323L565, 0721L710, 1221L440, 0622L675, and 0521L200) and were infected either IP or SC (subcutaneously) with CCHFV strains IbAr10200 (6–83 weeks of age) or Turkey04 (6 weeks of age). Mice were monitored daily for weight loss and clinical signs; clinical scoring and euthanasia were performed according to established protocols as previously described [20]. Samples were collected from serially euthanized mice infected with Turkey04 at 3, 7, 14, 21, and 28 days post-infection (dpi), and from mice infected with IbAr10200 at 3 dpi and after meeting euthanasia criteria (4–8 dpi). Group sizes were larger for the IbAr10200 analyses because historical unvaccinated/untreated control mice were included; fewer Turkey04-infected mice were available as historical controls.

### RT-qPCR

Urogenital swabs (preputial for males or intravaginal for females; sterile mini-tip polyester swab; Puritan, Cat. No. 25-800 1PD 50) collected for vRNA extraction were placed in 0.5 mL MagMAX lysis buffer and incubated at room temperature (RT) for 15–20 min. Reproductive tissues (∼100 mg section of testis or ovary and seminal vesicle or cervix) were collected, placed in 1.0 mL of MagMAX Lysis/Binding Solution Concentrate, and homogenized using the 2010 Geno/Grinder (SPEX SamplePrep). Total RNA was extracted and vRNA was quantified using methods previously described [19]. RNA levels were normalized using the validated reference genes *Ppia* and *Gusb* [20].

### Virus isolation and quantification

Urogenital swabs (preputial for males or intravaginal for females; sterile mini-tip polyester swab; Puritan, Cat. No. 25-800 1PD 50) collected for virus isolation were placed in 1.0 mL serum free DMEM (2× antimycotic/antibiotic; Gibco, Cat. No. 15240062) and incubated 15–20 min at RT. Samples were centrifuged to clear particulates, and supernatants were plated (250 µL/well) into 4 wells of a 12-well plate seeded with Huh7 cells. CCHFV (10-fold dilution series) and DMEM alone were plated in parallel as positive and negative controls. After rocking every 15 min for 1 h (37°C), DMEM (5% FBS and 2× antimycotic/antibiotic; Gibco, Cat. No. 15240062) was added to each well and plates were incubated at 37°C for 5 days. Plates were fixed and stained as previously described [19]. Results are reported as + or – detection of virus.

Reproductive tissues remaining after collection for RT-qPCR (males: 1× testis, seminal vesicle; females: 1× ovary, uterus) were homogenized in serum free DMEM (1.0 mL; 2× antimycotic/antibiotic; Gibco, Cat. No. 15240062), centrifuged, plated onto 96-well plates seeded with Huh7 cells (100 µL/well; 10-fold dilutions), and incubated at 37°C for 5 days. CCHFV (10-fold dilution series) and DMEM alone were plated in parallel as positive and negative controls. Plates were fixed and stained as above. Results are reported as TCID_50_/g using the Reed-Muench method [21].

### Pathology methods

Reproductive tissues (testis, epididymis, accessory sex glands for males; ovary, oviduct, uterus for females) were fixed in 10% neutral buffered formalin and processed for routine paraffin histology and hematoxylin–eosin staining [19]. Immunohistochemistry (IHC) and in situ hybridization (ISH) were performed on reproductive tissues from subsets of animals. IHC was performed using a rabbit anti-CCHFV N pAb (IBT Bioservices, Cat. No. 04-0011), as previously described [19]. To localize CCHFV vRNA in FFPE (formalin-fixed paraffin-embedded) tissues, ISH assays using an RNAscope 2.5 HD Red Detection Kit and CCHFV strain-specific probes for detection of NP transcripts (mRNA/cRNA) (IbAr10200 [V-CCHFV-S-N-C1] and Turkey04 [V-CCHFV-S], Advanced Cell Diagnostics; Cat. No. 1193601-C1 and 510621) were performed, as previously described [22]. All tissues were tested in parallel with appropriate positive and negative controls. Specificity testing for CCHFV probes were verified by testing of FFPE tissues from clinically or genetically similar viruses.

### Analytical approach and statistical analyses

The primary objective of this work was to link reproductive tissue data with disease outcome rather than route of exposure. Accordingly, pooled analyses were conducted across both inoculation groups, as tissue replication kinetics and distribution do not differ notably by exposure route [19]. Any route-specific differences observed are noted in the text. All statistical analyses were conducted using GraphPad Prism version 10.2.2. Mann–Whitney tests were used to compare mean vRNA levels across age groups and between samples that were positive or negative for infectious virus.

## Results

### CCHFV RNA and infectious virus are widely detected in mouse reproductive tissues in the absence of severe tissue pathology during lethal disease

**Figure 1.**
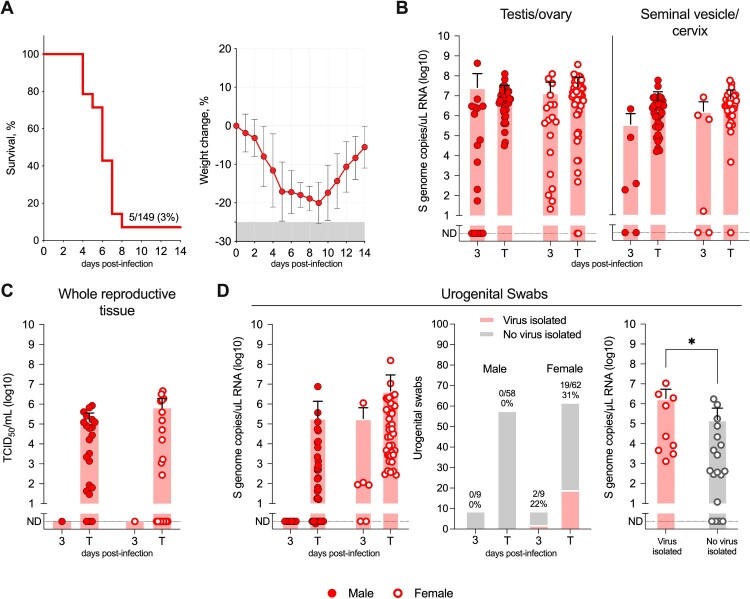
Groups of male and female C57BL/6J mice were transiently immunosuppressed with an anti-IFNAR1 monoclonal antibody (MAR1-5A3; intraperitoneally [IP]), infected with lethal CCHFV strain IbAr10200 (IP or subcutaneously [SC]), and serially euthanized at 3 days post infection (dpi) or at the terminal timepoint (meeting euthanasia criteria or succumbing to disease [T]). (A) Historical data from the CCHFV transient immunosuppression (IS) model [19] demonstrating that CCHFV IbAr10200-infected mice lose significant weight (>20% from baseline measured on day 0) and succumb to infection between 4 and 8 dpi (3% survival at 14 dpi, n = 5/149). (B) Tissues including gonad (1 × testis or ovary) and seminal vesicle or cervix (∼100 mg sections) were collected to determine levels of CCHF viral RNA (vRNA) via RT-qPCR. (C) Remaining reproductive tissues (males: 1 × testis, seminal vesicle; females: 1× ovary, uterus) were collected for evaluation of infectious CCHFV titre. (D) Urogenital swabs (preputial for males, intravaginal for females) were collected from each animal at the time of euthanasia for evaluation via RT-qPCR and virus isolation. Mann-Whitney tests were used to determine if mean vRNA copy number was different between samples that were positive or negative for infectious virus; * *p* < 0.5. ND, not detected.

C57BL/6J mice immunosuppressed with anti-IFNAR1 mAb MAR1-5A3 and infected (intraperitoneally [IP] or subcutaneously [SC]) with CCHFV strain IbAr10200 develop severe disease with substantial weight loss (>20% from baseline measured on day 0) resulting in lethality 4 to 8 days post infection (dpi) (3% survival at 14 dpi, n = 5/149) (Figure 1A) [19]. Here, groups of male and female mice were transiently immunosuppressed (mAb MAR1-5A3), infected (IP or SC) with IbAr10200, and serially euthanized at 3 dpi or at the terminal timepoint (meeting euthanasia criteria or succumbing to disease; see Methods). Urogenital swabs (preputial for males, intravaginal for females) were collected from animals at the time of euthanasia for vRNA detection by RT-qPCR and for virus isolation. Gonad (1 × testis or ovary) and seminal vesicle or cervix (∼100 mg section) were also collected to determine levels of vRNA via RT-qPCR. Remaining reproductive tissues (males: 1 × testis, seminal vesicle; females: 1 × ovary, uterus) were collected for evaluation of infectious CCHFV titre. Separate cohorts of infected mice were euthanized and whole reproductive tissues were collected for histopathological analysis by H&E staining. A subset of samples was also evaluated by immunohistochemistry and/or *in situ* hybridization.

Early after infection at 3 dpi, vRNA was detectable in the gonad and seminal vesicle/cervical tissue of a majority of both male (gonad: 13/20, 65%; seminal vesicle: 6/9, 66%) and female (ovary: 21/21, 100%; cervix: 7/9, 78%) mice. By terminal timepoints (4–8 dpi) vRNA was detectable in both tissues in 100% of male (45/45) and 96% of female (50/52) mice. Viral RNA copy number peaked at terminal timepoints averaging 1.3E+07 (range: 3.18E+04 to 1.26E+08) and 2.9E+07 (range: not detected [ND] to 3.71E+08) S genome copies/µL in the gonad (Figure 1B), and 4.7E+06 (range: 1.52E+04 to 5.72E+07) and 6.9E+06 (range: ND to 5.69E+07) S genome copies/µL in seminal vesicle/cervical tissue from male and female mice, respectively. In addition to vRNA, high titres of infectious CCHFV were also isolated from whole homogenized reproductive tissue of a majority of both male (mean: 1.30E+05 [range: ND to 8.45E+05] TCID_50_/g) and female (mean: 6.33E+05 [range: ND to 4.58E+06] TCID_50_/g) mice at terminal timepoints but, notably, could not be isolated at early timepoints (3 dpi) (Figure 1C). We also found that tropism for reproductive tissue did not change based on mouse age at the time of inoculation, which ranged from 6 to 83 weeks (terminal IbAr10200-infected mice only); mean vRNA levels were not significantly different across age groups (Supplemental Figure 1).

Viral RNA was frequently detected in urogenital swabs from female mice at 3 dpi and at terminal timepoints (3 dpi: 4/6, 67%; terminal: 40/40, 100%). In contrast, detection in males was less frequent (3 dpi: 0/6, 0%; terminal: 28/41, 68%), likely reflecting the superficial nature of preputial swabbing compared with the intravaginal collection used in females. High copy numbers of vRNA were detected in swabs from both males (mean: 1.99E+05 [range: ND to 7.56E+06] S genome copies/µL) and females (mean: 4.55E+06 [range: 2.74E+02 to 1.55E+08] S genome copies/µL) at terminal timepoints (Figure 1 D). Importantly, infectious virus could be isolated from the urogenital swabs of female mice at 3 dpi and terminal timepoints; none was isolated from males (Figure 1C). Higher vRNA values were associated with higher likelihood of isolation of infectious virus from urogenital swabs (Figure 1D).

**Figure 2.**
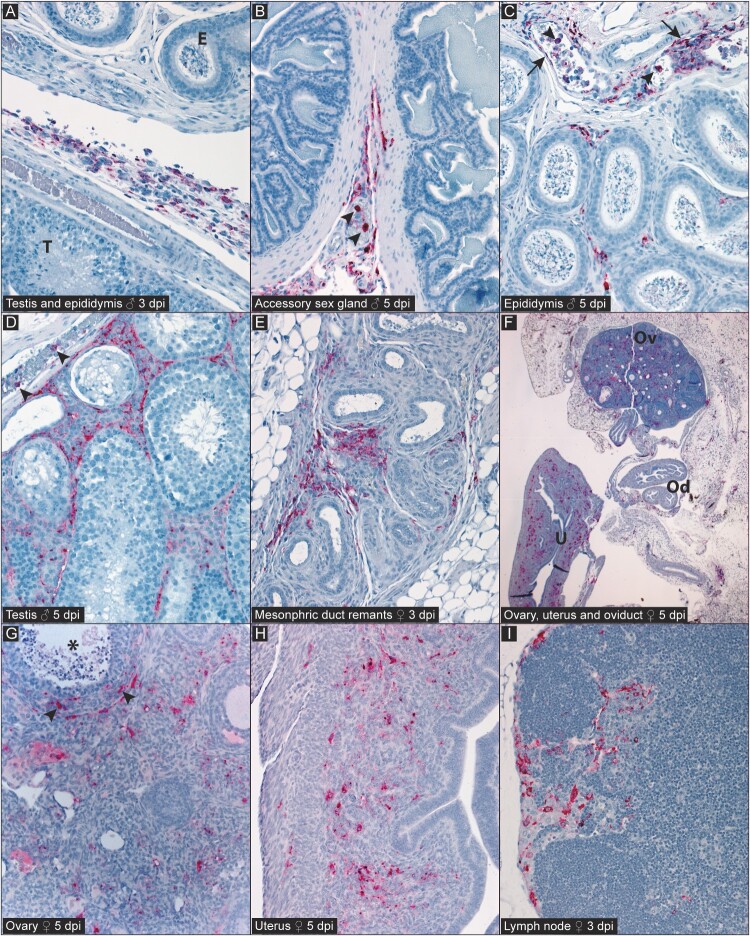
Detection of CCHFV antigen (red) by immunohistochemistry (IHC) in reproductive tissues of C57BL/6J mice infected with lethal strain IbAr10200 at 3 dpi or at the terminal timepoint (meeting euthanasia criteria or succumbing to disease; (representative images from 5 dpi). Note absence of mucosal epithelial staining in all tissues. [A] 3 dpi male with immunostaining in inflammatory infiltrate between the surfaces of the testis (T) and epididymis (E). [B] 5 dpi male with immunostaining in the serosal tissue of the accessory sex gland, including intravascular leukocytes (arrowheads). [C] 5 dpi male with immunostaining of the epididymal interstitium and fibrovascular connective tissue, including endothelial cells (arrows) and intravascular leukocytes (arrowheads). [D] 5 dpi male with immunostaining among interstitial cells and in intravascular leukocytes (arrowheads) in the testis. [E] 3 dpi female with immunostaining in mesenchymal cells associated with mesonephric duct remnants. [F] 5 dpi female with widespread immunostaining in the ovary (Ov), Uterus (U), and surrounding fibroadipose tissues; Oviduct (Od). [G] Higher magnification of ovary in [F] showing staining in the ovarian stroma and in thecal cells (arrowheads) of a tertiary follicle (*). [H] Higher magnification of uterus in [F] showing immunostaining within the endometrial and myometrial layers. [I] 3 dpi male with immunostaining of cells in the subcapsular sinuses of a reproductive tract-associated lymph node. Polyclonal anti-nucleoprotein (NP) CCHFV antibody, naphthol fast red chromogen. Original magnifications: × 20 [A-E, G-I]; × 2.5 [F].⁣

Infection with lethal IbAr10200 was characterized by histopathologic changes in reproductive tissues at 3 dpi and terminal timepoints (4–6 dpi), regardless of inoculation route, that included primarily serosal histiocytic or mixed inflammation, as well as occasional foci of necrosis or granulomatous/pyogranulomatous inflammation attributed at least in part to intraperitoneal (IP) injection of mAb (MAR1-5A3) and/or IP inoculation of virus (Figure 2). One terminal (5 dpi) female had necrotizing granulomatous inflammation with possible intralesional foreign material in the visceral fat, also attributed to IP injection. In males, there was also typically mild, mixed inflammation of the epididymal fat pad, and occasionally, of the epididymal or accessory sex gland interstitium. In females, mixed inflammation was present in adipose tissue around the oviduct and uterus.

### Immunohistochemistry confirms infection of reproductive tissues in mice with lethal disease

Immunohistochemistry (IHC) was performed on a representative subset of reproductive tissues from animals infected with lethal IbAr10200 (Figure 2). CCHFV antigen (nucleoprotein) was detected in reproductive tissues by IHC at 3 dpi for both SC and IP infection routes and was most extensive in the serosa of IP-inoculated mice. Overall staining was more widespread, but with a similar distribution, in tissues from mice reaching endpoint criteria (4–6 dpi). Antigen was primarily localized to serosal mesothelium and inflammatory cells, interstitial stromal and inflammatory cells, intravascular leukocytes, and endothelial cells. There was also staining of histiocytes in reproductive tract-associated lymph nodes. In males, there was staining of the tunica vaginalis and the testicular and epididymal interstitium. In females, staining was in the ovarian bursa, stroma, and thecal cells, in oviductal and uterine interstitium, and in the myometrium. There was no staining of mucosal epithelium in male or female reproductive tissues.

### Nonlethal and lethal CCHFV infections exhibit similar reproductive tissue tropism and pathology during acute disease

**Figure 3.**
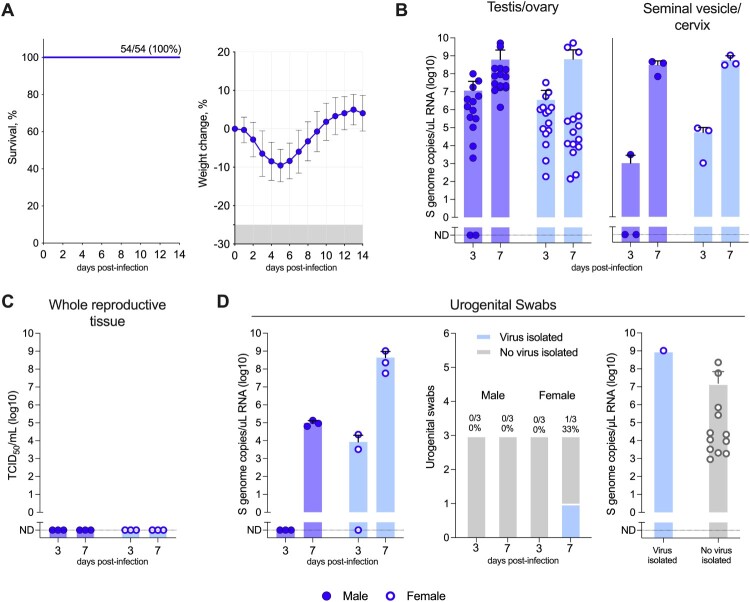
Groups of male and female C57BL/6J mice were transiently immunosuppressed with an anti-IFNAR1 monoclonal antibody (MAR1-5A3; intraperitoneally [IP]) infected with nonlethal CCHFV strain Turkey04 (IP or subcutaneously [SC]), and serially euthanized at 3- and 7-days post infection (dpi). (A) Historical data from the CCHFV transient immunosuppression (IS) model [19] demonstrating that CCHFV Turkey04-infected mice transiently lose weight (<15% from baseline measured on day 0) and all recover from infection (≥14 dpi) with no other signs of clinical disease (100% survival, n = 54/54). (B) Tissues including gonad (1 × testis or ovary) and seminal vesicle or cervix (∼100 mg section) were collected to determine levels of CCHFV viral RNA (vRNA) via RT-qPCR. (C) Remaining reproductive tissue (males: 1× testis, seminal vesicle; females: 1 × ovary, uterus) was collected for evaluation of infectious CCHFV titre. (D) Urogenital swabs (preputial for males, intravaginal for females) were collected from each animal at the time of euthanasia for evaluation via RT-qPCR and virus isolation. ND, not detected.

C57BL/6 mice immunosuppressed with mAb MAR1-5A3 and infected (IP or SC) with CCHFV strain Turkey04 exhibit mild weight loss (<15% from baseline measured on day 0) but no other signs of clinical disease, and all animals recover from infection (100% survival at ≥14 dpi, n = 54/54) (Figure 3A) [19]. Here, groups of mice were transiently immunosuppressed, infected (IP or SC) with Turkey04, and serially euthanized during acute infection, at 3 or 7 dpi. Samples collected from lethal IbAr1200-infected mice described above were similarly collected from Turkey04-infected mice.

During acute infection Turkey04 vRNA was detectable in both the gonad and seminal vesicle/cervix of most mice. At 3 dpi 87% (13/15) males and 100% (15/15) females had vRNA present in the gonad, and 67% (2/3) of males and 100% (3/3) females had vRNA present in the seminal vesicle/cervix. By 7 dpi vRNA was detectable in 100% of both tissues (gonad [15/15] and seminal vesicle/cervix [3/3]) in males and females. Viral RNA copy number peaked at 7 dpi, and mean titres (gonad, males: 6.21E+08 [range: 1.36E+06 to 5.14E+09], females: 6.53E+08 [range: 1.39E+02 to 5.19E+09] S genome copies/µL; seminal vesicle/cervix, males: 3.14E+08 [range: 7.11E+07 to 4.78E+08], females: 5.85E+08 [range: 3.11E+08 to 1.08E+09] S genome copies/µL) were comparable to those in the lethal IbAr10200 model (Figure 3B). In contrast to the lethal model, and despite high levels of vRNA, no infectious virus was isolated from reproductive tissues (Figure 3C).

Viral RNA was detectable in urogenital swabs early after infection at 3 dpi from female (2/3, 67%; mean: 8.4E+03 [range: ND to 2.19E+04] S genome copies/µL) but not male (0/3, 0%) mice. At 7 dpi 100% of urogenital swabs from both female (3/3) and male (3/3) mice had detectable vRNA; mean titres were significantly higher in females (4.38E+08 [range: 5.79E+07 to 1.03E+09] S genome copies/µL) compared to males (9.69E+04 [range: 6.38E+04 to 1.35E+05] S genome copies/µL). Rarely, infectious virus could be isolated from the urogenital swabs of female mice at 7 dpi (1/3, 33%). The single swab positive for infectious virus also corresponded to the highest level of vRNA detected in urogenital swabs (Figure 3D).

**Figure 4.**
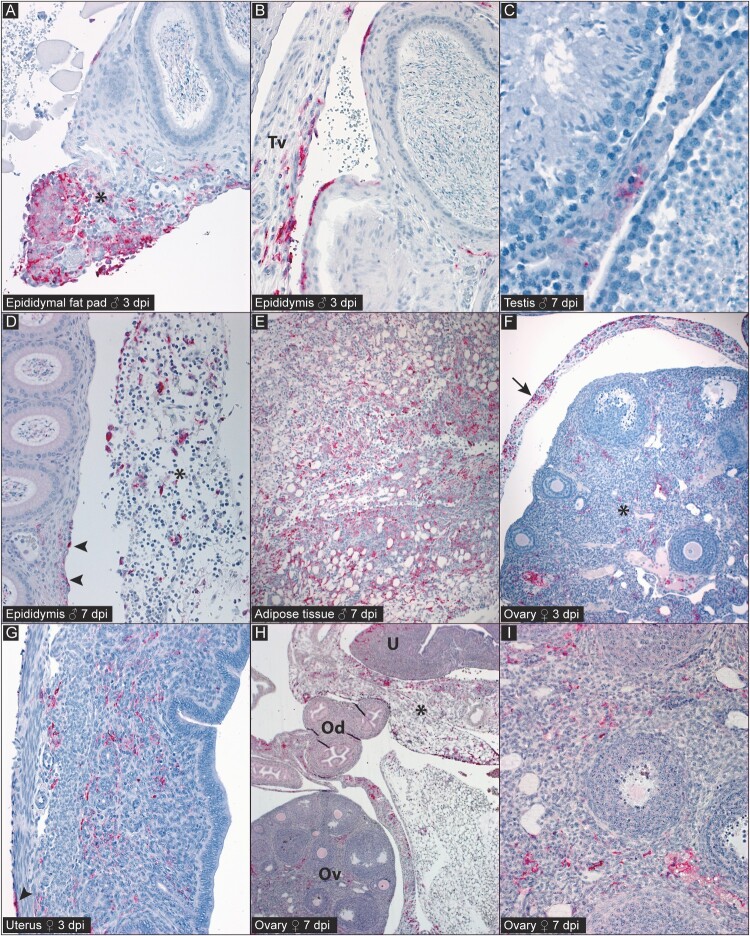
Detection of CCHFV antigen (red) by immunohistochemistry (IHC) in reproductive tissues of C57BL/6J mice infected with nonlethal strain Turkey04 in early infection (3 or 7 dpi). Note absence of mucosal epithelial staining in all tissues. [A] 3 dpi male with immunostaining in inflammatory infiltrate (*) in the epididymal fat pad. [B] 3 dpi male with immunostaining along the outer wall of the epididymal tubules and in the tunica vaginalis (Tv). [C] 7 dpi male with focal immunostaining among interstitial cells in the testis. [D] 7 dpi male with immunostaining along the surface of the epididymis (arrowheads) and in the adjacent inflamed connective tissue (*). [E] 7 dpi male with widespread immunostaining in densely inflamed adipose tissue surrounding reproductive tissues. [F] 3 dpi female with immunostaining in the ovarian parenchyma (*) and bursa (arrow). [G] 3 dpi female with immunostaining in the uterine endometrium, myometrium, and serosa (arrowhead). [H] 7 dpi female with widespread immunostaining in the ovary (Ov) and bursa, Oviduct (Od), outer myometrium of the uterus (U), and the surrounding adipose tissue (*). [I] Higher magnification of ovary in [H] showing immunostaining in the ovarian stroma. Polyclonal anti-nucleoprotein (NP) CCHFV antibody, naphthol fast red chromogen. Original magnifications: × 20 [A, B, D, E, G, I]; × 40 [C], × 10 [F], × 5 [H].

Histopathologic changes and viral antigen localization in reproductive tracts of Turkey04-infected mice at 3 and 7 dpi were similar to those observed in lethal IbAr10200-infected mice (Figure 4). Findings were characterized predominately by mixed inflammation of serosal surfaces and visceral connective tissues, including pyogranulomatous inflammation, which was again more prominent in IP-inoculated animals. There was again occasional involvement of parenchymal interstitium by mild, mixed inflammation. Viral antigen was localized by IHC to the same tissues and cell types as in lethally infected mice. There was also a lack of epithelial staining in male and female tissues, except for in one terminal (7 dpi) male that had epithelial staining in discrete segments of the head of the epididymis.

### CCHFV is largely cleared from reproductive tissues during convalescence

To investigate whether CCHFV could be detected in the reproductive tissues of survivors after convalescence, C57BL/6J mice infected with Turkey04, as previously described, were serially euthanized after recovery from mild acute disease (defined as weight loss) at 14, 21, and 28 dpi. One IbAr10200-infected female that recovered from severe disease and survived to 14 dpi was also included in histopathologic analyses.

**Figure 5.**
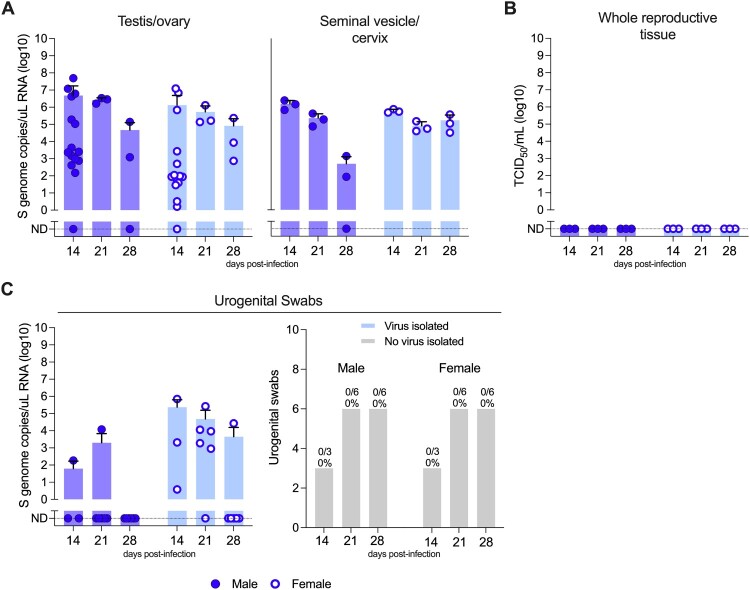
Groups of male and female C57BL/6J mice were transiently immunosuppressed with an anti-IFNAR1 monoclonal antibody (MAR1-5A3; intraperitoneally [IP]), infected with nonlethal CCHFV strain Turkey04 (IP or subcutaneously [SC]), and serially euthanized at 14-, 21-, and 28 days post infection (dpi). (A) Tissues including gonad (1 × testis or ovary) and seminal vesicle or cervix (∼100 mg section) were collected to determine levels of CCHFV viral RNA (vRNA) via RT-qPCR. (C) Remaining reproductive tissue (males: 1 × testis, seminal vesicle; females: 1 × ovary, uterus) was collected for evaluation of infectious CCHFV titre. (D) Urogenital swabs (preputial for males, intravaginal for females) were collected from each animal at the time of euthanasia for evaluation via RT-qPCR and virus isolation. ND, not detected.

In surviving Turkey04-infected mice, vRNA decreased incrementally between 14 and 28 dpi but remained detectable at 28 dpi in the gonad of males (mean: 4.67E+04 [range: ND to 1.39E+05] S genome copies/µL) and females (mean: 8.15E+04 [range: 7.45E+02 to 2.35E+05] S genome copies/µL), and in the seminal vesicle of males only (mean: 4.88E+02 [range: ND to 1.38E+03] S genome copies/µL) (Figure 5A). Interestingly, levels of vRNA did not significantly decrease in the cervical tissue of female mice between 14 (mean: 5.90E+05 [range: 4.82E+05 to 7.49E+05] S genome copies/µL) and 28 dpi (mean: 1.68E+05 [range: 3.20E+04 to 3.62E+05] S genome copies/µL). Urogenital swabs were rarely positive for vRNA in male mice at 14 (1/3, 33%) and 21 (1/6, 17%) dpi, and vRNA was no longer detectable at 28 dpi (0/6, 0%). In female mice, urogenital swabs were positive for vRNA through 28 dpi but frequency decreased over time (14 dpi: 3/3 [100%], 21 dpi: 5/6 [83%], 28 dpi: 1/6 [17%]). Infectious virus was not isolated from reproductive tissues or urogenital swabs of convalescent animals (Figure 5B-C).

**Figure 6.**
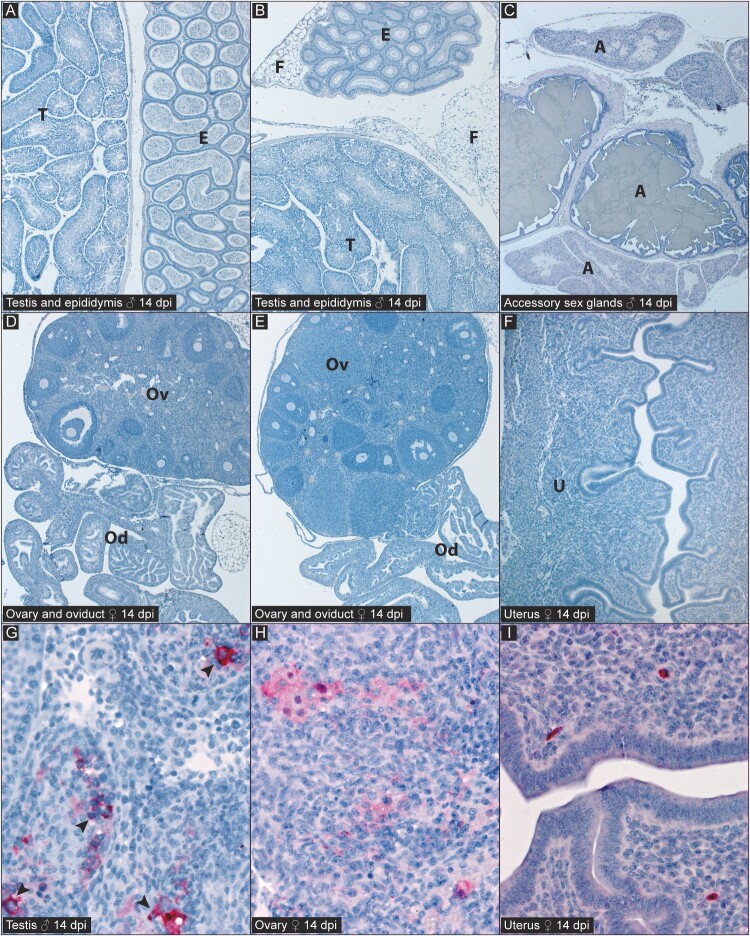
Immunohistochemistry (IHC) for CCHFV antigen (red) in reproductive tissues of convalescent C57BL/6J mice infected with strain Turkey04 [A-G] or IbAr10200 [H-I], 14 dpi. [A-C]: Reproductive tissues from 14 dpi males infected with CCHFV strain Turkey04 show absence of viral antigen in testes (T), epididymides (E), epididymal fat (F), and accessory sex glands (A). [D-F] Reproductive tissues from 14 dpi females infected with CCHFV strain Turkey04 show absence of viral antigen in ovaries (Ov), oviducts (Od), and uterus (U). [G] Testis from 14 dpi male infected with CCHFV strain Turkey04 shows focal staining of inflamed seminiferous tubules, including apparent seminiferous epithelium (arrowheads). [H, I] Reproductive tissues from the single survivor of CCHFV strain IbAr10200 infection with rare immunostaining in the ovary [H] and uterus [I] at 14 dpi. Polyclonal anti-nucleoprotein (NP) CCHFV antibody, naphthol fast red chromogen. Original magnifications: × 5 [A-F]; × 40 [G-I].

Among Turkey04-infected mice at 14 dpi, mild chronic inflammation was occasionally present in serosal and interstitial tissues, but CCHV antigen was not detected (Figure 6A-F) except focally in the testes of two males, where it localized to a cluster of inflamed seminiferous tubules, including apparent spermatogenic precursors (Figure 6G). Tissues from the one IbAr10200-infected female that survived to 14 dpi had focal lymphocytic infiltrates in the periovarian connective tissue and scattered lymphocytes in the myometrium. Antigen localized to the inflammatory infiltrate around mesonephric duct remnants in the periovarian fat, and focally within the ovarian stroma and rare individual endometrial stromal cells (Figure 6H-I). No viral antigen was detected in reproductive tissues from Turkey04-infected survivors at 21 or 28 dpi.

### In situ hybridization using strain-specific probes is more sensitive than IHC for CCHFV detection and localization in tissues

**Figure 7.**
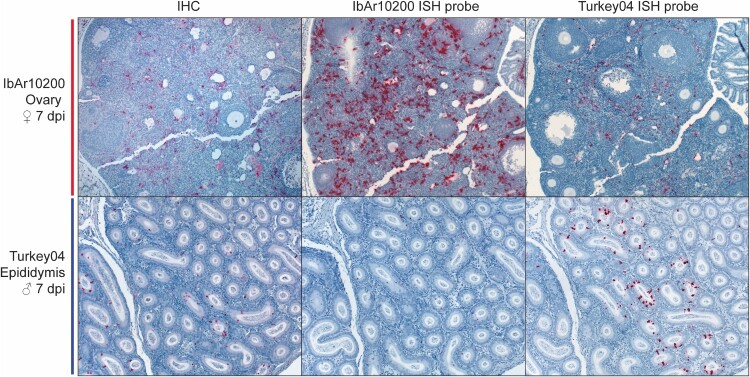
Comparison of immunohistochemistry (IHC) and in situ hybridization (ISH) for detection and localization of CCHFV in formalin-fixed, paraffin-embedded C57BL/6J mouse reproductive tissues. Top row: ovary from a mouse inoculated with CCHFV strain IbAr10200, 7 dpi. Bottom row: epididymis from a mouse inoculated with CCHFV strain Turkey04, 7 dpi. Left column: IHC using anti-CCHFV NP pAb, naphthol fast red chromogen. Middle column: ISH using IbAr10200-specific probe to detect NP mRNA/cRNA. Right column: ISH using Turkey04-specific probe to detect NP mRNA/cRNA. Epididymis from a mouse inoculated with CCHFV-Turkey04 shows strongest staining by Turkey04-specific ISH, while ovary from a mouse inoculated with CCHFV-IbAr10200 shows strongest staining by IbAr10200-specific ISH. IHC shows less staining than the matched-strain ISH but more staining than the opposite-strain ISH for both tissues. Note that epididymis shown in the bottom row has prominent staining in tubular epithelial cells and is the only tissue from any animal in the study that had mucosal epithelial staining. Original magnifications: × 10.

To maximize sensitivity and confirm that IHC methods did not miss low levels of CCHFV that may be present during convalescence, in situ hybridization (ISH) assays were developed to investigate the cellular localization of CCHFV mRNA/cRNA in mouse reproductive tissues. The assays were performed systematically on tissues from a subset of animals to compare the sensitivities of ISH and IHC for CCHFV detection in formalin-fixed paraffin-embedded (FFPE) tissues. ISH using strain-specific probes (i.e. IbAr10200 or Turkey04) showed similar overall distribution, but more abundant staining and sometimes differences in cellular localization within individual tissues than IHC in reproductive tissues from mice infected with the corresponding strain. ISH using heterologous strain probes showed less extensive staining than both ISH using homologous strain probes and IHC (Figure 7). Despite this increased sensitivity of strain-specific ISH compared to IHC, staining by ISH was not identified in any tissue section that did not also have some, albeit less, staining by IHC, for tissues that had both assays performed.

### Discussion

Here, we evaluated CCHFV loads (vRNA and infectious titre) in reproductive tissues and urogenital swabs from both lethal (strain IbAr10200) and survivor (strain Turkey04) mouse models of CCHF [19]. Viral RNA in reproductive tissues was detected in at least a subset of mice at all timepoints examined, irrespective of virus strain, with the highest levels detected during acute disease (3 and 7 dpi) or at terminal endpoint (4–8 dpi). In urogenital swabs, evaluated as a proxy for virus transmission, vRNA was detected in animals infected with both IbAr10200 or Turkey04; it was first detected in females (3 dpi) and present in all mice at 7 dpi (Turkey04) or at terminal sampling (IbAr10200). Viral RNA levels in reproductive tissues and urogenital swabs from terminal IbAr10200-infected mice were consistent across age groups and advanced age, known to be a risk factor for Ebola virus persistence in human survivor semen, was not associated with higher vRNA levels [23].

In tissues, infectious virus was only isolated from IbAr10200-infected mice at terminal timepoints. However, high levels of vRNA were also detected during mild disease after infection with Turkey04, suggesting that while isolation may be rare in these animals, additional studies with larger cohorts are warranted to more accurately determine the frequency of infectious virus recovery. Like tissues, virus isolation from urogenital swabs was more frequent following lethal IbAr10200 infection than nonlethal Turkey04 infection. Infectious virus was recovered only from intravaginal swabs at 7 dpi (Turkey04) or terminal sampling (IbAr10200); no virus isolates were obtained from males, sampling was limited to preputial surface swabs which may not fully reflect shedding risk.

In both lethal and nonlethal models, immunostaining during acute infection was primarily associated with inflammation of visceral connective tissues and adjacent serosal surfaces, and within the parenchymal interstitium, which was often only minimally inflamed. Serositis in these animals was interpreted to be due at least in part to intraperitoneal inoculation of virus (for IP-inoculated animals) and/or IP injection of mAb (all animals). Viral antigens and RNA localization by IHC and ISH, respectively, within serosal infiltrates of both IP- and SC-inoculated animals supports the possibilities of viral dissemination to the serosa by both direct inoculation and through viremia or leukocyte trafficking. Mucosal epithelium appeared generally spared in both males and females, with segmental epididymal epithelial staining seen in only one male (7 dpi), and no mucosal epithelial staining seen in any female reproductive tissue. These findings are consistent with prior reports in female IFNAR ^-/-^ mice infected with CCHFV IbAr10200 which demonstrated focal staining in the lamina propria of the terminal oviduct and the ovarian medullary stroma [18].

In other viral infections where sexual transmission is known to occur, such as Zika virus, infection of germ cells and epididymal epithelium are well characterized [24,25]. Interestingly, our findings are more consistent with findings from EBOV-infected mice and macaques during acute infection, where epithelial cell staining is rare and antigen is primarily detected in reproductive tract interstitium, stromal connective tissue, and macrophages, rather than parenchymal cells [26,27]. These data suggest that rare epithelial cell infection during CCHF does not preclude the possibility of sexual transmission, and even uncommon findings in disease models may translate into substantial public health consequences, including severe disease and resurgent outbreaks, as have been reported with EBOV transmission from survivors [6,9]. These data also support that the mechanism of sexual transmission for viral hemorrhagic fevers may be different than those traditionally described for other viruses, and further work is needed to elucidate this mechanism.

In convalescent animals, no virus was isolated from swabs or tissues despite continued detection of vRNA until 28 dpi, suggesting low risk for persistence of infectious virus and sexual transmission during convalescence. However, rare immunostaining was observed in a small subset of animals at 14 dpi. Notably, in two male convalescent mice, antigen distribution differed from that seen during acute infection, with staining present in seminiferous tubules in addition to the interstitium. This raises the possibility that, although uncommon, direct infection of spermatogenic precursors may occur. These findings parallel observations in nonhuman primates infected with EBOV, a virus known to persist in reproductive tissues, where interstitial staining is the primary finding after convalescence [28]. Thus, while we cannot exclude the potential for viral shedding in a subset of animals, its apparent rarity indicates that confirmation would require investigation with larger cohorts.

Given the large numbers of human CCHF cases and survivors each year, even rare shedding from the reproductive tract of survivors could have significant implications for sexual transmission [1,2]. Importantly, tissue localization and isolation of infectious virus from urogenital swabs in both models suggest the potential for sexual transmission during acute infection, even during mild disease. Urogenital swab vRNA levels corresponded with likelihood of isolating infectious virus, underscoring the value of investigating this association in human patients. If validated, vRNA detection from urogenital swabs could be used clinically to assess transmission risk in CCHF patients prior to hospital discharge. Ultimately, structured survivor programmes for CCHF, modelled after those established for EBOV [29,30], would be highly valuable.

Limitations of this work include smaller cohort sizes for non-lethal infection, particularly for acute urogenital swabs and convalescent tissues and swabs, which reduced our ability to detect rare instances of infectious virus. In addition, urogenital swab sampling in males was less invasive, likely leading to an underestimation of shedding in this sex. Finally, detection of vRNA or infectious virus in tissues and swabs does not provide conclusive evidence of direct sexual transmission, though it does indicate a potential risk. In this study, we focused on indices of viral presence and infectivity in association with infection outcome, without considering host factors that may also influence replication kinetics and transmission risk. In previous work examining these mouse models, we demonstrated that proinflammatory cytokines and chemokines were significantly elevated in non-survivors, implicating inflammatory responses in disease outcome [19]. Such responses, together with other factors, including underlying conditions, are likely to affect both the incidence and duration of viral persistence in human disease.

While the evidence for CCHFV sexual transmission in humans remains anecdotal, it is notable that a single report from 1968 provided the only evidence of filovirus sexual transmission (Marburg virus) prior to the 2016 and 2021 EBOV outbreaks, where sexual transmission played a critical role [6,10,11,31]. The findings herein underscore the need for further research on human CCHFV infection to critically assess the risk of sexual transmission and the potential for viral persistence in reproductive tissue during and after disease resolution and identify optimal measures to monitor patients and mitigate subsequent exposure.

## Supplementary Material

Repro Manuscript_Main text_resub_v4_Marked.docx

Supplemental Figures_CCHFV Repro.docx
