# Supplementary material for: Crimean-Congo hemorrhagic fever virus localization and shedding in the reproductive tract of lethal and survivor mouse models
Source: Emerg Microbes Infect. 2025 Nov 27;15(1):2595795. doi: 10.1080/22221751.2025.2595795 (PMC12777786; doi:10.1080/22221751.2025.2595795)
Supplement: Supplemental Figures_CCHFV Repro.docx [file TEMI_A_2595795_SM6580.docx]

**Crimean-Congo Hemorrhagic Fever Virus Localization and Shedding in the Reproductive Tract of Lethal and Survivor Mouse Models**

Teresa E Sorvillo^1*^, Jana M. Ritter^2^, Stephen R. Welch^3^, Katherine Davies^3^, JoAnn D. Coleman-McCray^3^, Heather M. Hayes^2^, Georgia Ficarra^2^, Julu Bhatnagar^2^, Scott D. Pegan^4^, Éric Bergeron^3^, Joel M. Montgomery^3^, Christina F. Spiropoulou^3^, Jessica R. Spengler^3*^

**Affiliations:**

^1^ CDC Foundation assigned to Viral Special Pathogens Branch, Division of High Consequence Pathogens and Pathology, Centers for Disease Control and Prevention, Atlanta, GA, USA

^2^ Infectious Diseases Pathology Branch, Division of High Consequence Pathogens and Pathology, Centers for Disease Control and Prevention, 1600 Clifton Road, Atlanta, GA, USA

^3^ Viral Special Pathogens Branch, Division of High Consequence Pathogens and Pathology, Centers for Disease Control and Prevention, 1600 Clifton Road, Atlanta, GA

^4^ Division of Biomedical Sciences, University of California Riverside, Riverside, CA, USA

**Supplemental Figure 1.** Mouse age does not alter viral RNA levels in reproductive tissues and urogenital swabs from CCHFV IbAr10200-infected mice at terminal timepoints.

**
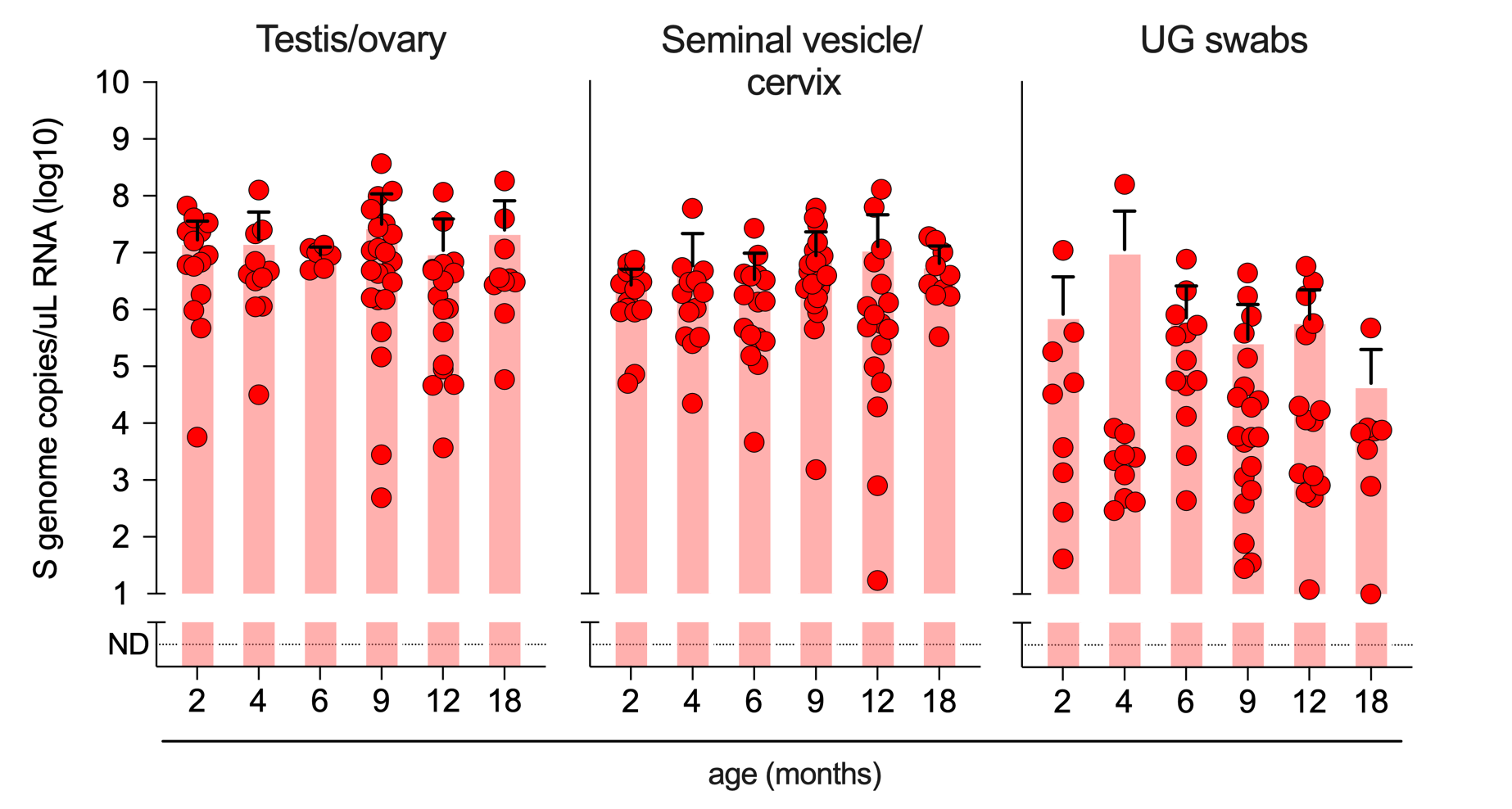
**

**Supplementary Figure 1.** Mouse age does not alter viral RNA (vRNA) levels in reproductive tissues and urogenital swabs from CCHFV IbAr10200-infected mice at terminal timepoints. Groups of male and female C657BL/6 mice ranging in age from approximately 2 to 18 months were transiently immunosuppressed with an anti-IFNAR1 monoclonal antibody (MAR1-5A3; intraperitoneally [IP]) infected with CCHFV strain IbAr10200 (IP or subcutaneously [SC]) and euthanized when meeting euthanasia criteria (see Methods). Tissues including gonad (1 × testis or ovary) and seminal vesicle or cervix (~100 mg section) were collected to determine levels of CCHFV vRNA via RT-qPCR. Urogenital swabs (UG; preputial for males, intravaginal for females) were also collected from each animal at the time of euthanasia for evaluation via RT-qPCR. Mann-Whitney tests were used to compare mean vRNA levels across age groups; no significant differences were identified.
